# Genetic control of Group 3 (K96) capsule synthesis and complement resistance in extraintestinal pathogenic *Escherichia coli*

**DOI:** 10.1128/msphere.00237-26

**Published:** 2026-07-10

**Authors:** Rachael J. David Prince, Rajesh Bogati, Titan Lytle, Andres Collado Silva, Rachael A. LeBaron, Tiana Sorge, Michael A. Olson, Eric Wilson, David L. Erickson

**Affiliations:** 1 Department of Microbiology and Molecular Biology, Brigham Young University 6756, Provo, Utah, USA; 2 Snow College 3233, Ephraim, Utah, USA; US Food and Drug Administration, Silver Spring, Maryland, USA

**Keywords:** capsule, ExPEC, phase variation, oxyR, mastitis-associated *E. coli*, complement resistance

## Abstract

Extraintestinal pathogenic *Escherichia coli* (ExPEC) frequently produce Group 2 or 3 capsules that enhance virulence, yet genetic regulation and specific roles of Group 3 capsules remain poorly understood. Here, we demonstrate that the K96 Group 3 capsule in ExPEC strain M12 confers resistance to human serum complement. Using genetic screens, we identified phase-variable mutations in *kpsC* and essential roles for transcriptional regulators OxyR and RfaH in activating capsule synthesis via a distant promoter. Mutants lacking OxyR or RfaH failed to produce capsule, exhibited complement sensitivity, and showed attenuated virulence in *Galleria mellonella*. These findings reveal novel mechanisms controlling Group 3 capsule expression and underscore the capsule’s contribution to immune evasion.

**IMPORTANCE:**

Group 2 capsules are established extraintestinal pathogenic *Escherichia coli* (ExPEC) virulence factors and are the type most often associated with human isolates. Group 3 capsules have previously been considered a sub-type of Group 2, although nothing was known about factors controlling their expression. Capsule serotype K96-encoding ExPEC strains are increasingly isolated from human infections and inhabit numerous other hosts. It is critical to understand the factors that enable their pathogenic versatility and survival in specific environments. Our study shows that the K96 capsule of strain M12 is required for human complement resistance. Additionally, we have identified genetic factors that control Group 3 capsule synthesis, including a potential phase-variable mechanism as well as transcriptional control by OxyR. Co-regulation of Group 3 capsule synthesis genes with genes necessary for oxidative stress resistance may increase the virulence of some versatile ExPEC strains.

## INTRODUCTION

Extraintestinal pathogenic *Escherichia coli* (ExPEC) are a leading cause of serious human diseases, including pneumonias and bloodstream infections. Approximately 240,000 yearly deaths are attributable to bloodstream infections due to these bacteria (1). Many invasive infections begin as a urinary tract infection (UTI), and ~60% of women experience at least one UTI caused by *E. coli* (2). ExPEC also causes diseases of livestock and companion animals, and their zoonotic potential is increasingly recognized (3–6). Expanding resistance to antimicrobial drugs greatly complicates their treatment (7–9). Individual ExPEC strains may possess a variety of virulence determinants, including metal acquisition systems, adhesins, toxins, and protective structures that shield them from host immune defenses, including lipopolysaccharide O-antigen and capsular polysaccharides. However, there are no genetic markers that reliably differentiate ExPEC from commensals or intestinal pathogens (10).

Surface polysaccharides, including K antigen capsules, enhance the virulence of many *E. coli* strains (11). More than 90 different K antigens are classified into four broad groups based on their mode of assembly and gene arrangement (12, 13). Groups 1 and 4 capsules are polymerized in the periplasm and exported in a Wzx/Wxy-dependent manner, while Groups 2 and 3 are polymerized at the cytoplasmic face of the inner membrane and exported via ABC transporters (14). Specific capsules have diverse functions, including protecting bacteria from serum complement, inhibiting phagocytosis, forming intracellular bacterial communities, resisting antimicrobial peptides, and avoiding detection by macrophage-encoded receptors (15–18).

Most capsules made by ExPEC belong to Group 2 or 3 (19–23), and previous investigations have focused on the Group 2 capsules. Group 3 *kps* loci contain genes necessary for initiation (*kpsS*, *kpsC*) and export (*kpsD*, *kpsM*, *kpsT*, *kpsE*) of the glycolipids that comprise these capsules, as well as genes for serotype-specific glycosyltransferases that polymerize the sugar subunits. KpsS and KpsC add ketodeoxyoctonic acid to a phospholipid carrier, upon which the capsule is built in the cytoplasm prior to export by the ABC transport apparatus (KpsDMTE). While the synthesis, export, and biological functions of Groups 2 and 3 capsules are presumed to be similar, their genetic organization at the *kps* locus differs. There are two opposing transcriptional units for Group 2 capsules, whereas Group 3 capsule genes appear to be arranged in a single operon. Group 2 capsule loci contain *kpsF* and *kpsU* genes that are absent from Group 3, suggesting that they are not needed or that their functions are fulfilled by genes found elsewhere in the genome. A model for Group 2 capsule expression and regulation has been developed (24), which includes genes encoded outside of the *kps* locus necessary for synthesis (25–27). In contrast, beyond the fact that they are not temperature-dependent, Group three capsule regulation is not understood (13).

We recently showed that *E. coli* strain M12, originally isolated from a clinical case of bovine mastitis, is highly virulent in mouse models of mammary gland infection, ascending urinary tract infection, and intraperitoneal sepsis (28). Strain M12 belongs to the ST69 lineage, like many human ExPEC strains emerging worldwide (29). This suggests that M12 and perhaps other mastitis-associated strains have the potential to cause ExPEC-like disease in humans. However, whether strain M12 or other mastitis-associated isolates can resist human innate immune defenses has not been examined. M12 produces a Group 3 capsule, which is critical for survival in mouse kidneys during UTI and intraperitoneal infections (28), and moderately enhances fitness in mammary gland infections (30). This capsule belongs to serotype K96 (31) and likely contains d-glucuronic acid and l-rhamnose (32). K96 is similar to the K54 capsule, produced by the human ExPEC strain CP9, which contains glucuronic acid and rhamnose modified with threonine or serine (33). K96 and K54 capsules are sufficiently similar that anti-K54 antiserum also binds the K96 capsule on strain M12 (28). The K54 capsule of strain CP9 is important during soft tissue and bloodstream infections, but unlike the M12 capsule, it does not affect survival during UTI in either the bladder or the kidneys (34). The K54/96 capsules most commonly occur in strains belonging to the ST69 lineage (like M12), and a recent rapid expansion of human bloodstream isolates that have acquired these Group 3 loci has been documented (35).

While our previous work demonstrated that the unencapsulated M12 Δ*kpsCS* mutant is severely attenuated in some tissues, we did not determine the basis for the virulence defect, and specific functions for K96 capsules more broadly are unknown. Some ExPEC capsules confer protection against serum complement, while others do not (36–39), and the effect of capsule on complement-mediated killing likely depends on other factors that vary among ExPEC strains. Although M12 requires capsule to survive in mouse kidneys and the peritoneal cavity, mice are not reliable as a model host to study the role of complement and bacterial resistance factors for human pathogens (40). In this study, we sought to determine whether the K96 capsule affects M12 survival in human blood and serum, and to identify genetic and environmental factors that affect Group 3 capsule synthesis.

## RESULTS

### M12 capsule confers resistance to human serum complement

**Fig 1:**
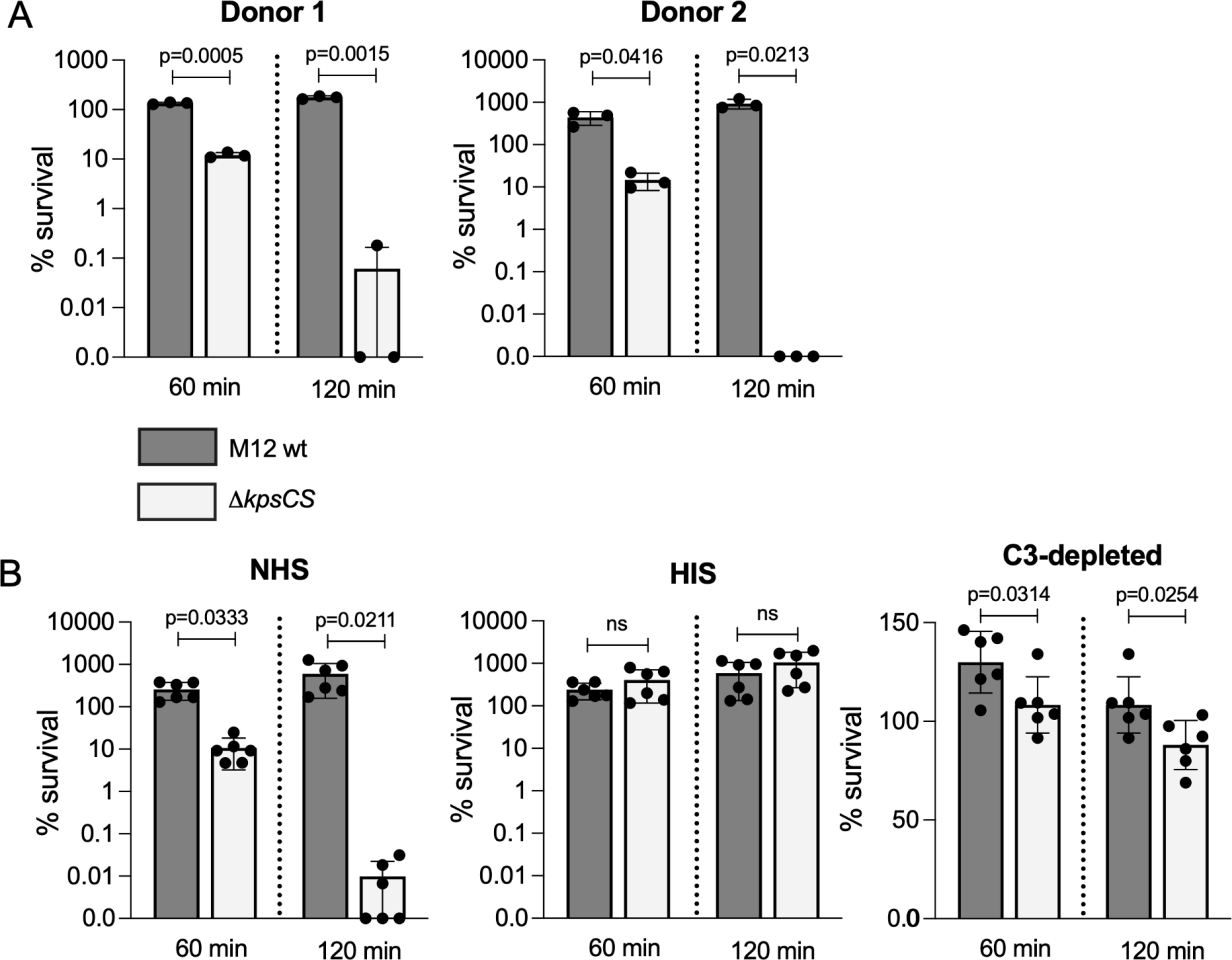
Group 3 K96 capsule confers resistance to human blood and serum complement in strain M12. (**A**) Survival of M12 wild-type and Δ*kpsCS* mutant in whole human blood obtained from two different donors. Individual replicates (*n* = 3) for each donor are represented, and error bars represent the standard deviation. (**B**) Survival of M12 wild-type and Δ*kpsCS* mutant in pooled normal human serum (NHS), heat-inactivated serum (HIS), and C3-depleted serum, *n* = 6 for each. *P* values were calculated using Welch’s *t*-test.

To investigate strain M12 as a potential human pathogen and define the role of its K96 capsule, we first determined its survival in whole human blood. Strain M12 grew very well in blood obtained from two different donors, increasing up to 10-fold over a 2-h period (Fig. 1A). The Δ*kpsCS* mutant strain was significantly more sensitive to killing in whole blood compared to the M12 wild-type strain and was substantially reduced (>3-log decrease) or not recovered after 120 min of incubation.

Next, we compared survival of the wild-type M12 strain with that of the Δ*kpsCS* mutant in pooled normal human serum (NHS) and in heat-inactivated serum (HIS) (Fig. 1B). While the wild-type strain replicated in both NHS and HIS, the Δ*kpsCS* mutant in NHS was rapidly killed. HIS did not kill either strain, suggesting that the complement was responsible for the death of the mutant strain. We also tested bacterial survival in serum depleted of the complement C3 protein (Fig. 1B). Like HIS, C3-depleted serum had very little effect on survival of either the wild-type or Δ*kpsCS* mutant strain, although the wild-type M12 CFU counts were consistently slightly higher than the mutant (Fig. 1B).

### Identification of genes required for K96 capsule synthesis

Capsule synthesis can reduce the density of bacterial cells, which allows separation of encapsulated from unencapsulated bacteria using Percoll gradients (41). A gradient consisting of 55% and 75% Percoll separated the encapsulated wild-type M12 strain from the unencapsulated Δ*kpsCS* mutant cells, with the unencapsulated bacteria found in the bottom layer (Fig. S1). We used this approach to separate rare, unencapsulated mutants in a previously generated M12 transposon library (30). In a preliminary small-scale screen, single colony-forming units were recovered from the bottom layer of Percoll gradients (putatively containing unencapsulated bacteria). Alcian blue staining was used to confirm a lack of capsule production in 26 of these mutants (data not shown); the location of the transposon insertion sites was then determined by arbitrary PCR. This revealed fifteen unique transposon insertion sites (Table 1), including *kpsD*, *kpsT*, *kpsE*, *kpsS*, *kpsC*, *rmlC*, and putative glycosyltransferase genes within the *kps* locus, suggesting that this approach could be used at a larger scale to identify relevant genes. We also obtained nine mutants with insertions outside of the *kps* region (Table 1), representing genes whose function has not been associated previously with capsule production. To verify that these nine genes outside the *kps* region were involved in capsule production, we attempted to complement each mutant with functional copies of the disrupted gene on high-copy plasmids. This restored capsule synthesis, at least to some extent, in the *yjhH*::Tn5 and *ispA*::Tn5 mutants (Fig. S1), indicating that these two genes are involved in capsule production. However, the other seven mutants outside of the *kps* locus were not complemented by this approach.

**TABLE 1:** Genes disrupted in unencapsulated Tn5 isolated from Percoll gradients

Gene name	No. of unique insertions	Function	Capsule restored by complementation
*kpsD*	2	Capsule biosynthesis/transport protein	Not tested
*kpsT*	1	Capsule ABC transporter ATP-binding protein	Not tested
*kpsE*	3	Capsule export inner membrane protein	Not tested
*wcaA*	4	Putative glycosyltransferase	Not tested
*wsaE*	1	Putative glycosyltransferase	Not tested
*rmlC*	1	dTDP-4-dehydrorhamnose 3,5-epimerase	Not tested
*kpsC*	3	Capsule biosynthesis/initiation protein	Not tested
*kpsS*	2	Capsule biosynthesis/initiation protein	Not tested
*ispA*	1	Farnesyl diphosphate synthase	Yes
*arnC*	1	Undecaprenyl-phosphate 4-deoxy-4-formamido-l-arabinose transferase	No
*yjhH*	1	Putative 2-dehydro-3-deoxy-d-pentonate aldolase	Yes
*mlaC*	1	Outer membrane asymmetry protein	No
*yahL*	1	Hypothetical protein	No
*ybfL*	1	Transposase	No
*oleD_1*	1	Oxoalkanate reductase	No
*rbfX*	1	O-antigen transport	No
*yahK*	1	Aldehyde reductase	No

**Fig 2:**
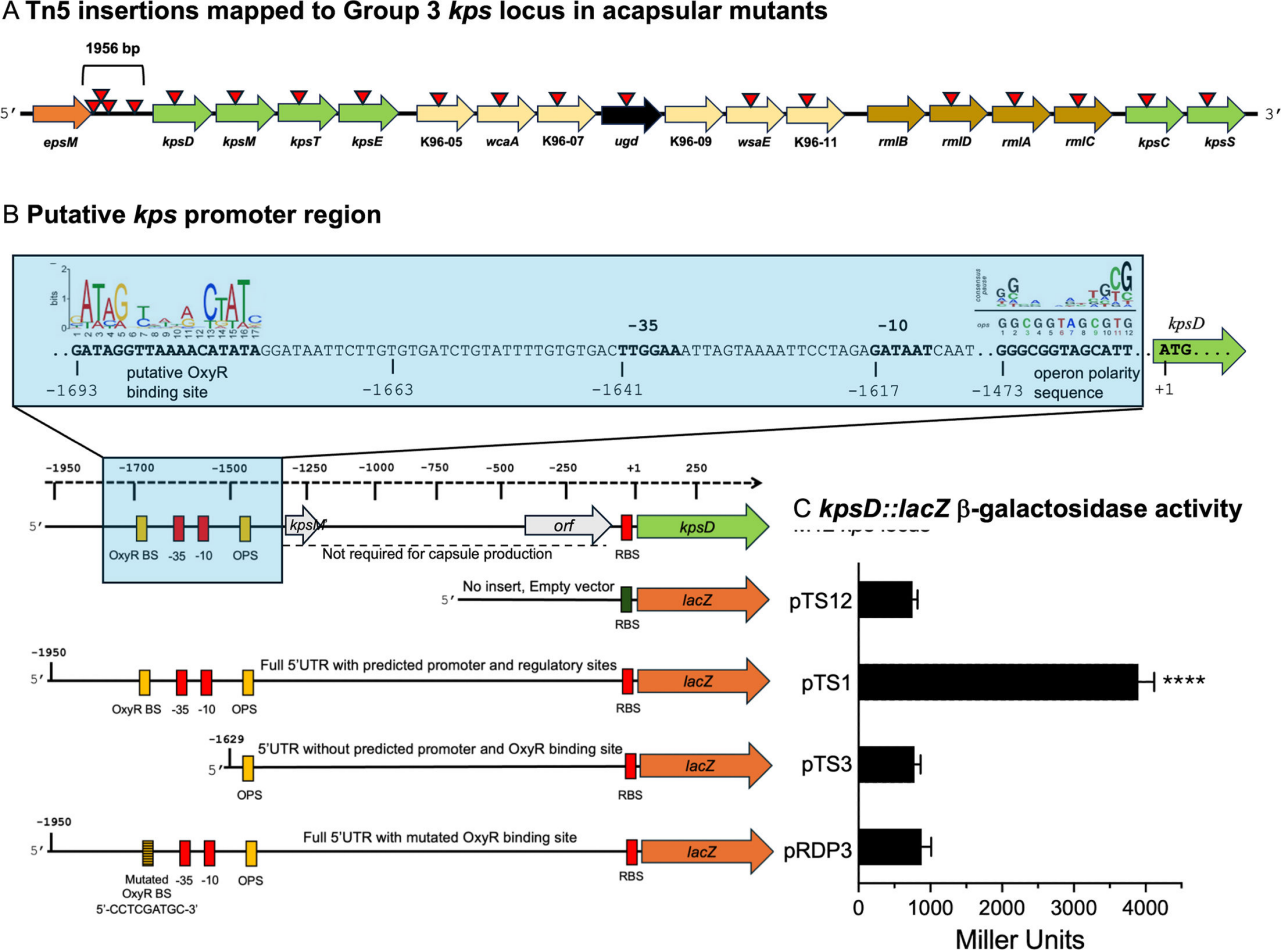
Genes within the *kps* locus required for capsule synthesis, including potential *kpsD* promoter region. (**A**) Structure of the *kps* locus, including those that were identified in the TnSeq analysis as necessary for capsule production, indicated with triangles. Many insertions in the unencapsulated transposon mutant population mapped to the intergenic region between *kpsD* and the type II secretion (T2SS) gene *epsM*. (**B**) Promoter features identified within the T2SS-*kpsD* intergenic region, including putative −35/−10 regions ~1,600 bp upstream of *kpsD*. An operon polarity sequence (ops) necessary for RfaH-mediated transcription of long operons is located 1,473 bp upstream of *kpsD*. The consensus ops element is included for comparison. A putative OxyR-binding site 5′ to the −35/−10 sequence was identified by comparison with the consensus binding sequence from reference 42. (**C**) *kpsD::lacZ* β-galactosidase assays demonstrate that the predicted promoter region and 5’ UTR, including the OxyR-binding site, activate transcription (pTS1), whereas a fragment that omits the putative −35/−10 region (pTS3) does not. Mutation of the OxyR-binding site (pRDP3) results in activity equal to the empty vector control plasmid (pTS12). ****, P value < 0.0001 by Student’s *t*-test compared with pTS12.

Following this small-scale, proof-of-principle screen, we next undertook a larger-scale approach to identify additional genes required for capsule synthesis. Percoll separations were used to enrich for unencapsulated mutants present in the transposon mutant library without isolating single colonies, followed by high-throughput sequencing of the transposon junctions. The transposon junctions for the total (unselected) library were also sequenced as controls. The insertion sites were mapped to the M12 genome. As expected, reads from the unencapsulated bacteria mapped to relatively few sites compared with the unselected samples (Table S1). Genes overrepresented in the unencapsulated fraction relative to the entire library represent those potentially necessary for capsule synthesis (log fold change > 2 and *P* value < 0.05). According to this analysis, all genes in the *kps* locus except one predicted glycosyltransferase (*k96-09*) and *rmlB* appear to be required for capsule synthesis (Fig. 2A). Putative capsule-associated genes outside of the *kps* locus include several involved in carbohydrate transport and metabolism, hydrogenases, and transcriptional regulators such as OxyR and RfaH (Table S2).

Mapping of the transposon sequencing reads to the M12 genome revealed that many of the transposon insertions in the capsule-negative population occurred within the 1,956 nucleotide region between *kpsD* and type II secretion genes (Fig. 2B). There is a *kpsM*′ pseudogene fragment that is not predicted to yield a protein, as well as a hypothetical open reading frame just upstream of *kpsD* within this region. We deleted this region (−1450 to −50 relative to *kpsD*) from the chromosome of strain M12. This mutant produced capsule (data not shown), demonstrating that neither the *kpsM*′ fragment nor the hypothetical protein is required. We also located potential −35 and −10 promoter sigma factor binding sites 1,641 and 1,617 nucleotides from the KpsD start codon (Fig. 2B). There is an operon polarity sequence (ops) necessary for RfaH-mediated transcription elongation (43) downstream of the predicted −35 and −10 promoter site. We also identified a putative OxyR-binding site 5′ to the predicted −35 and −10 promoter site (Fig. 2B).

To determine whether this region could activate transcription, we created a reporter plasmid with the 1,950-nucleotide *kpsD* upstream region fused to the *lacZ* gene. This fragment was sufficient to induce β-galactosidase activity in strain DH5a, which lacks native β-galactosidase. However, when the putative promoter and OxyR-binding sites were omitted from the reporter plasmid, β-galactosidase activity decreased to levels similar to the plasmid-only control (Fig. 2C). We also created a plasmid that retained the promoter, but the putative OxyR-binding site was altered, which also resulted in β-galactosidase expression indistinguishable from the plasmid-only control (Fig. 2C). These results suggest an unusually long 5′ untranslated region in the *kps* operon and that OxyR may be required for activating Group 3 capsule gene expression in strain M12.

### RfaH and OxyR are required for Group 3 capsule production

**Fig 3:**
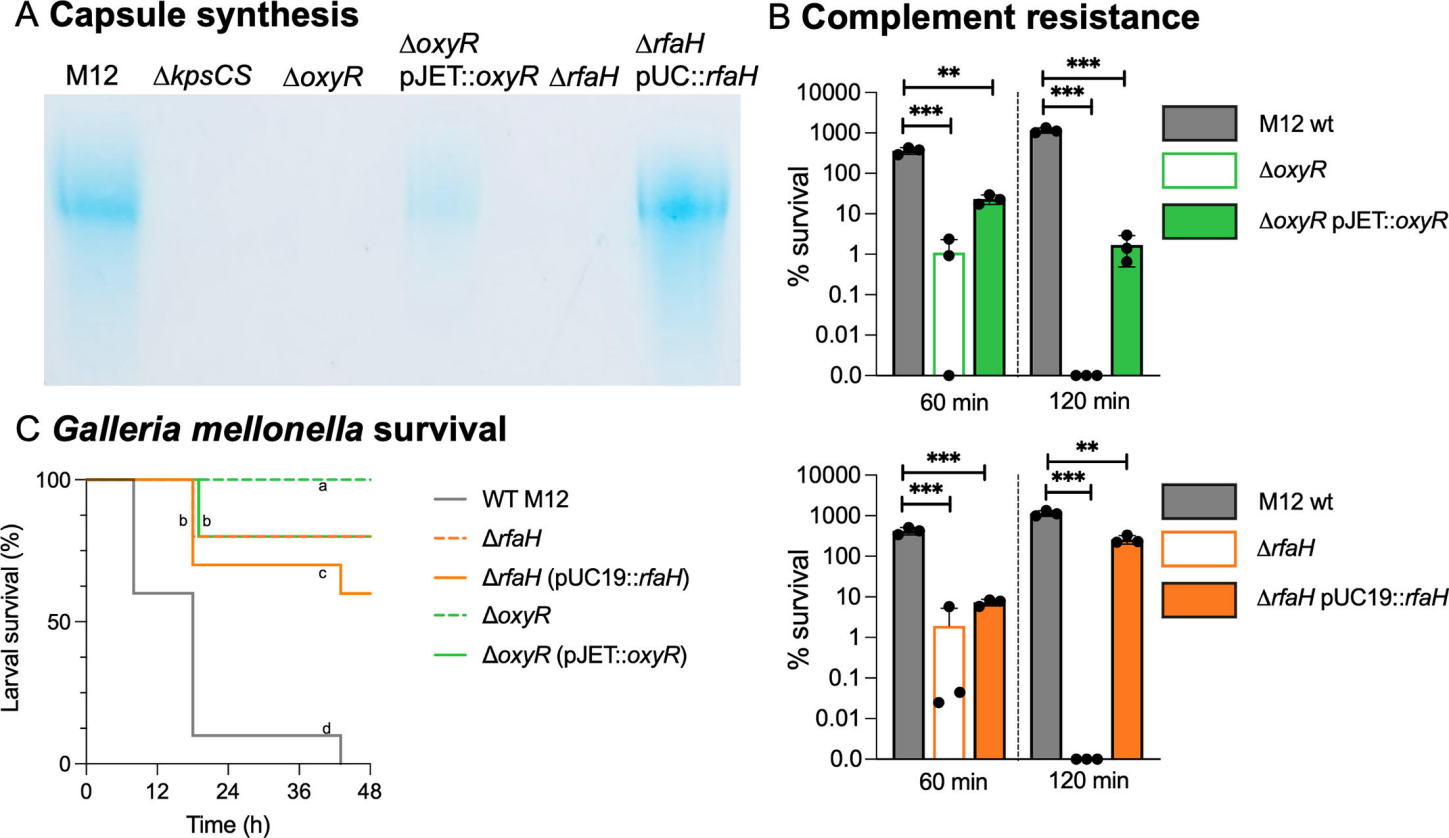
RfaH and OxyR are required for Group 3 K96 capsule production, serum survival, and virulence in *G. mellonella*. (**A**) Capsule production in wild-type M12, Δ*kpsCS,* Δ*rfaH*, Δ*oxyR*, and complemented mutants was detected by SDS-PAGE and Alcian blue staining. (**B**) Bacterial survival of the same strains incubated in NHS. Asterisks indicate a statistically significant difference from the wild-type strain at the given time by Dunnett’s multiple comparison test, **, P value < 0.01; ***, P value < 0.001 (**C**) Survival of *G. mellonella* larvae infected with 10^4^ CFU of the same strains. Different letters indicate significant differences between strains (log-rank test, *P* < 0.05 after Sidak’s correction).

The TnSeq results and the presence of an ops element downstream of the potential transcriptional start site suggested that RfaH may be required for Group 3 capsule synthesis. We confirmed this hypothesis by creating a Δ*rfaH* mutant strain, which failed to produce capsule. Capsule production was restored in the mutant complemented with the functional *rfaH* gene on a plasmid (Fig. 3A). TnSeq and reporter assays also indicated a requirement for OxyR to produce the K96 capsule. We deleted *oxyR* from strain M12, and this mutant strain also failed to produce capsule (Fig. 3A). The complemented Δ*oxyR* mutant strain produced capsule, although at visibly lower levels than the wild-type strain (Fig. 3A).

Since capsule is required for complement resistance, we tested whether the Δ*rfaH* and Δ*oxyR* mutant strains survive in NHS. Consistent with their role in capsule synthesis, both mutants were very sensitive to NHS and were not recoverable after 2 h (Fig. 3B). The complemented *rfaH* mutant did not survive as well as wild-type M12 in NHS but was more resistant than the mutant strain. The complemented *oxyR* mutant strain also displayed intermediate sensitivity to serum, consistent with its incomplete restoration of capsule synthesis. Capsule is also required for the virulence of strain M12 in *Galleria mellonella* infections (28). When compared to the wild-type M12, both the Δ*rfaH* and Δ*oxyR* mutant strains were extremely attenuated in this infection model (Fig. 3C). The complemented *rfaH* mutant was significantly more virulent than the mutant strain, and the complemented *oxyR* mutant was also significantly different from the mutant in its ability to kill *G. mellonella*. However, both complemented mutant strains remained attenuated compared to the wild-type M12. This may be due to copy number effects of these regulators or issues related to the timing of gene expression that do not fully resemble the wild-type strain.

### Spontaneous genetic variation leading to loss of Group 3 capsule

**Fig 4:**
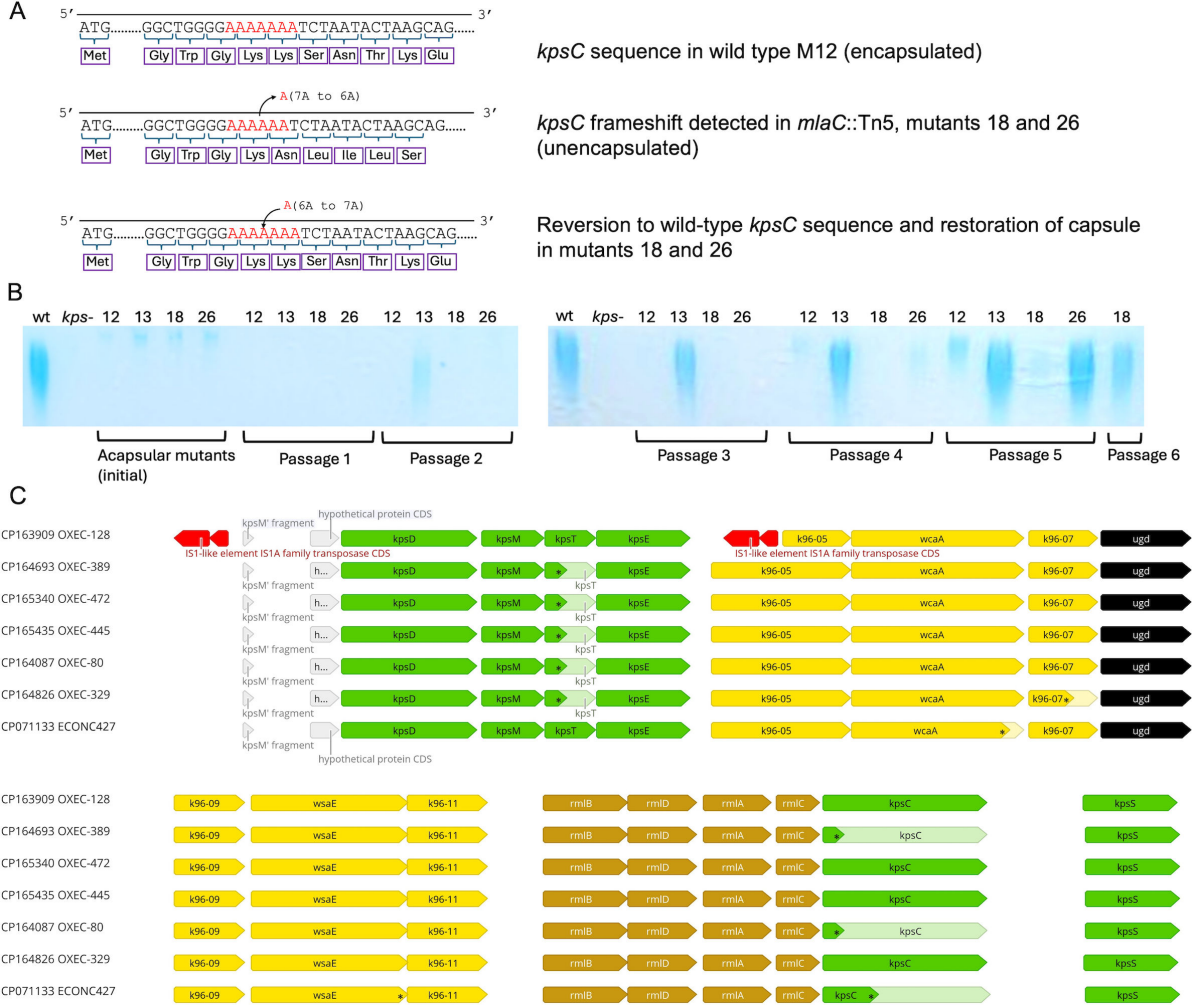
Some spontaneous mutations causing capsule loss are reversible. (**A**) Deletion identified within a homopolymeric site of adenosines in *kpsC* in an *mlaC*::Tn5 transposon mutant as well as in two spontaneous mutants derived from M12 wild-type parent strain (see Table 2). (**B**) Cultures of four spontaneous mutants (12, 13, 18B, and 26A; see Table 2) were passed through Percoll gradients, the top portion removed to recover any capsule-producing revertants, and then subcultured. The gradient separations were repeated once each day for 6 days, and the capsule production within the population was assessed by Alcian blue staining. Extracts from the wild-type M12 strain and Δ*kpsCS* mutant were included on each gel as controls. Capsule reversion in the population derived from mutant 13 was detectable after two passages, whereas reversion appeared by passage 4 in mutants 12 and 26, and passage 5 for mutant 18 (which was further enriched by passage 6). (**C**) Mutations predicted to inactivate capsule synthesis in other isolates containing K96 loci. IS element insertion in 5′ UTR and k96-05 (OXEC-128), and single-nucleotide deletions/insertions within *kpsT* (OXEC-389, OXEC-445, OXEC-80, and OXEC-329), *wcaA* (ECON427), k96-07 (OXEC-329), *wsaE* (ECON427), and *kpsC* (OXEC-389, OXEC-80, and ECON427) are predicted to inactivate capsule production. The same 7A-6A reversible frameshift mutation within *kpsC* that was observed in M12 occurs in OXEC-389 and OXEC-80.

As noted above (Table 1; Fig. S1), complementation failed to restore capsule synthesis in seven transposon mutants with insertions in genes outside the *kps* locus. This could be due to polar effects of the transposon insertions or non-optimal expression of the relevant genes from the high-copy plasmids. Among the non-*kps* transposon mutants, we were particularly interested in the *mlaC* gene because of its role in outer membrane asymmetry. Therefore, we inserted the entire *mlaFEDCB* operon on a low-copy plasmid into the *mlaC*::Tn5 mutant, and this also failed to restore capsule synthesis. We generated a new D*mlaC* deletion mutant, which produced capsule like the wild-type M12 strain (data not shown), showing that the Mla system is not required for capsule production. We then sequenced the genome of the *mlaC*::Tn5 transposon mutant to determine if it harbored additional mutations (multiple transposon insertions or polymorphisms) that affect capsule synthesis. In addition to the Tn5 insertion in *mlaC*, we identified a single-nucleotide deletion within *kpsC* at a repetitive section of adenosines (7A to 6A), resulting in a frameshift mutation (Fig. 4A), explaining the lack of capsule production in the *mlaC*::Tn5 mutant.

Frameshift mutations at homopolymeric sites can facilitate variable expression of capsules in other bacteria (44, 45). Therefore, we sought to estimate the number of unencapsulated bacteria present in saturated wild-type M12 cultures and investigate the genetic basis for their appearance. When grown in LB, the proportion of the cells that were recovered from the bottom of the gradient was 0.0043% ± 0.0036% of the total bacteria in the tube. In comparison, when the bacteria were grown in LB containing 2% bile salts, which we previously showed causes loss of capsule (28), 12.99% ± 9.17% were found in the bottom layer. We observed that most single colony-forming units recovered from the bottom fraction resumed capsule synthesis upon regrowth, suggesting transient loss of capsule gene expression rather than inactivating mutations. However, we isolated 16 distinct mutants that did not produce capsule upon regrowth in LB media. Sequencing revealed that all but one of these mutants contained a mutation within the *kps* locus, including single-nucleotide deletions, substitutions, IS629 insertions, and larger deletions (Table 2). Two independently derived mutants (18B and 26A) possessed the identical deletion within the homopolymeric site in *kpsC* (Table 2). Two mutants (12 and 13) had IS629 insertions in different genes. To determine if *kpsC* frameshift and IS insertion mutations are reversible, we added a barcoding plasmid to these four mutants to prevent and detect contamination and repeated the Percoll separations, this time recovering and subculturing the top portion of the gradient (Fig. 4B). Mutant 13 regained detectable capsule production after two passages. After four passages, we also detected capsule production in the populations derived from mutants 12 and 26A. After five passages, we had recovered capsule-producing revertants derived from each of the barcoded mutants. For the frameshift mutants (18B and 26A), resequencing showed that the homopolymeric site in *kpsC* had been restored in the revertants. Collectively, this suggests that Group 3 capsule production may be regulated by the regular appearance of inactivating mutations, including via IS elements and rare slipped-strand mispairing in *kpsC*. To determine whether this occurs naturally, we retrieved all the *E. coli* genome sequences that contain complete K96 loci from NCBI (58 total strains, Fig. S2). Most (51) of them are complete, with SNPs present throughout that may or may not impact capsule synthesis positively or negatively. However, five strains were identified that contained an identical single-nucleotide deletion within *kpsT*, one strain with an IS element within *k96-05*, and one with single-nucleotide insertions/deletions in *wcaA*, *wsaE*, and *kpsC* (Fig. 4C). Thus, at least 12% of these isolates likely do not produce the K96 capsule. Notably, two strains show the identical 7A to 6A frameshift mutation in *kpsC* that we obtained in our experiments with M12.

**TABLE 2:** Mutations identified in spontaneous unencapsulated mutants^*a*^

Mutant	Gene	Predicted function	Nature of mutation	Effects^*b*^
1	*wsaE*	Glycosyltransferase	Single-nucleotide substitution	W128*
3	*wcaA*	Glycosyltransferase	Single-nucleotide deletion	Frameshift
12	*ugd*	UDP-glucuronic acid synthesis	IS629 insertion	Gene disruption
13	*wsaE*	Glycosyltransferase	IS629 insertion	Gene disruption
15A	*wcaA*	Glycosyltransferase	Single-nucleotide substitution	H125Y
16B	*rbsR*	Ribose operon repressor	Single-nucleotide substitution	A241V
	*mreB*	Cell shape determining protein	Single-nucleotide substitution	P115L
17A	*wcaA*	Glycosyltransferase	Single-nucleotide insertion	Frameshift
18B	*kpsC*	Capsule initiation	Single-nucleotide deletion^*a*^	Frameshift
19A	*wcaA*	glycosyltransferase	Single-nucleotide deletion	Frameshift
20A	*kpsC*	Capsule initiation	Single-nucleotide insertion	Frameshift
21A	*wsaE*	Glycosyltransferase	Single-nucleotide substitution	W383C
22A	*kpsS*	Capsule initiation	Single-nucleotide substitution	I814F
23A	*wcaA*	Glycosyltransferase	Four-nucleotide deletion	Frameshift
24A	*kpsC*	Capsule initiation	Single-nucleotide substitution	L675*
	*irp*1	Non-ribosomal peptide/polyketide synthase HMWP1	Single-nucleotide insertion	Frameshift
25A	*gspC2-*k96-11	Type II secretion and *kps* locus genes	25,700-nucleotide deletion	Gene loss
26A	*kpsC*	Capsule initiation	Single-nucleotide deletion^*a*^	Frameshift

^*a*^ Identical mutations in tandem A repeat region.

^*b*^ *, stop codon.

## DISCUSSION

In this study, we demonstrated that strain M12 is resistant to human serum complement if it expresses its K96 capsule (Fig. 1). Although it was isolated from a case of bovine mastitis, these findings and previously reported experiments in animal models of UTI and sepsis (28) suggest the possibility that it could cause invasive disease in humans or other animals. Strains whose genomes are extremely similar to M12 have been isolated from a variety of human infections and other environments, further supporting this claim (Table S3). Strains that cause bovine mastitis have not previously been considered potential human pathogens. Although rarely possessing genes encoding Group 2 or 3 capsules (46), mastitis-associated *E. coli* strains are often highly resistant to bovine serum (47), which likely increases the severity of mammary gland infections and invasive disease in cattle. Further work is needed to understand the mechanisms controlling complement resistance in this group of bacteria. They often carry virulence genes contributing to adherence, iron acquisition, and immune evasion and carry out an ascending infectious process similar to UTI (48). Their relevance as sources of human disease will likely increase in tandem with the unfortunate popularization of unpasteurized milk and other dairy products.

ExPEC capsules play important roles in evading host defenses (15, 17, 18, 49). Genetic variation within the *kps* gene clusters is substantial, and some K types are strongly associated with invasiveness (35). These variations emerge due to horizontal gene transfer and recombination (50). K96 capsules are associated with high invasiveness among bloodstream ExPEC isolates (35), although the molecular basis is unknown. The precise mechanism whereby the K96 capsule confers serum resistance remains to be determined. One strong possibility is that it recruits the control protein Factor H, which inactivates spontaneously generated C3b at the cell surface (51) to prevent activation of the alternative pathway. The glucuronic acid in the K96 capsule confers a polyanionic structure and therefore might recruit Factor H (52), similar to the purported function of sialic acid-containing capsules such as K1 (53). It is also possible that the K96 capsule does not promote C3 inactivation but rather sequesters the membrane attack complex far from its target. In this case, the deposition of complement proteins could still opsonize the bacteria, leading to enhanced phagocytic killing. K96 capsule could also shield antibody targets important to the activation of the classical pathway and opsonization. Further experiments are required to distinguish between these possibilities.

The extreme sensitivity of the M12 Δ*kpsCS* mutant to complement was somewhat unexpected, as M12 also contains the gene cluster encoding O-antigen belonging to serotype O15. This O-antigen is sufficient to confer significant resistance to bovine serum in mastitis clinical isolate P4 (54), but apparently not in strain M12. It is possible that strain M12 produces only rough LPS, or that the disparate outcomes are related to differences between bovine and human complement.

There are certain environments wherein maintaining at least a fraction of unencapsulated cells would be beneficial, such as in the presence of some phages or capsule-targeting antibodies. We have previously shown that growth of encapsulated M12 bacteria in the presence of bile salts is delayed, likely because of insufficient metal uptake (28). Downregulation or inactivation of capsule synthesis could also potentially improve attachment to specific receptors or surfaces. Mechanisms that ensure capsule phenotypic diversity would therefore be expected. We readily isolated spontaneous unencapsulated M12 mutants with a variety of changes within the *kps* locus (Table 2). This included isolates with IS insertions or with a frameshift mutation in a homopolymeric site in *kpsC*, which we showed were reversible (Fig. 4). This finding underscores the caution that should be taken in interpreting transposon mutant phenotypes, as demonstrated by the *mlaC*::Tn5 mutant that also had a *kpsC* frameshift mutation. Several of the mutants we isolated (Table 2), as well as naturally occurring strains (Fig. 4), have IS elements disrupting the *kps* operon. Similar IS-mediated capsule inactivation has been noted for *Klebsiella pneumoniae* strains in gut environments (55, 56). These mutants may be better adapted to survival in the intestines but would be eliminated in some extraintestinal sites. Future studies should address whether Group 2 or 3 capsule genes in ExPEC strains are more broadly frequently inactivated or undergo phase variation and whether the fitness of capsule-negative M12 strains is altered during intestinal colonization. Wider sampling from diverse sources could determine whether the other specific mutations we identified in M12 (Table 2) arise in other strains.

Of the 17 genes within the M12 K96 locus (Fig. 2), 15 were predicted by TnSeq to be required for capsule production, including all of the conserved Group 3 *kps* genes. One gene that the analysis suggested may not be required was *k96-09*, encoding a probable glycosyltransferase. There are six predicted glycosyltransferases, the precise functions of which are not known. The transposon library had few insertions in the *k96-09* gene (30), potentially masking its importance. The *rmlB*, which encodes dTDP-glucose 4,6-dehydratase, also may not be required. To produce K96 antigen, UDP-glucose dehydrogenase (*ugd*) must generate UDP-glucuronic acid, and dTDP-rhamnose must be produced by the *rmlBDAC* gene products. The *rmlB* gene contained many insertions in both the original library and the unencapsulated fraction. The same enzyme is also encoded within the *wec* locus in the M12 genome for producing enterobacterial common antigen (57). This redundant gene could be sufficient for capsule synthesis.

Our study also identified other capsule-related genes beyond the *kps* locus. We confirmed *ispA* to be necessary for capsule synthesis (Table 1; Fig. S1). IspA synthesizes farnesyl diphosphate, which is a precursor to undecaprenyl phosphate (58). Undecaprenyl phosphate is important for building peptidoglycan, lipopolysaccharide, and some types of capsule polysaccharides (59). However, Groups 2 and 3 capsules are assembled in the cytoplasm on a phosphatidylglycerol lipid anchor (13). It is possible that the ABC transport apparatus needed to produce K96 antigen is unable to function without *ispA*. The 2-dehydro-3-deoxy-d-pentonate aldolase YjhH is also necessary. Neither of these genes was detected in the TnSeq analysis, suggesting that our screen was not saturating and additional genes could be identified. Other genes that were identified in both the preliminary (*rbfX* and *arnC*) and TnSeq (*arnC*) screens were related to LPS synthesis. Changes in cell density in these mutants may have been caused by altered LPS structure or synthesis rather than capsule production, but further research is required to determine if this is the case. Another limitation of the study is that the capsule status of individual mutants and complemented strains was determined by Alcian blue staining, which is a non-specific dye that could also target other non-K96 polysaccharides. If those polysaccharides were of similar size as the K96 antigen, this could lead to a false-positive result.

Similar to Group 2 capsules (25), we showed that the transcriptional antiterminator RfaH is required for Group 3 capsule synthesis in strain M12. More surprisingly, our study revealed that *oxyR* is required for Group 3 capsule production, which has not been previously reported for any *E. coli* capsules. OxyR belongs to the LysR family of DNA-binding transcriptional regulators and responds to reactive oxygen species. These oxidize specific cysteine residues, resulting in the formation of a tetramer of OxyR subunits, and binding to a conserved DNA element. This binding activates oxidative defense genes like *katG*, *aphCF*, *oxyS*, *gorA*, and *dps* (42) to neutralize the threat. OxyR-mediated Group 3 capsule regulation is reminiscent of typhoid Vi capsule (60), which is also assembled in the cytoplasm and exported by an ABC-transport system (13), and in which polymorphisms arise frequently (61). OxyR also activates other virulence-associated factors that do not have obvious oxidative-stress resistance roles, including Ucl fimbriae carried by some ExPEC (62). Our experiments showed that aΔ*oxyR* mutant was unable to produce capsule (Fig. 3). Furthermore, when the putative OxyR-binding site was altered, the reporter plasmid containing the *kpsD* promoter yielded less β-galactosidase activity (Fig. 2C). While this is suggestive of direct OxyR-binding and transcriptional activation, this remains to be definitively shown. Regardless, OxyR-mediated direct or indirect activation may be a key feature that distinguishes Group 2 from Group 3 capsules. The ability to activate capsule synthesis alongside oxidative stress resistance genes may enhance the resistance of M12 and other Group 3 capsule-producing strains to phagocytes.

In summary, the K96 capsule confers high levels of resistance to human complement but may be detrimental in other environments, which may select for frequent capsule gene inactivation. In the context of extraintestinal infections, Group 3 K96 capsule genes do not dramatically affect M12 colonization of bladders or mammary glands (28, 30). However, initial colonization of the glands results in a strong influx of neutrophils to the lumen, which play critical roles in limiting infection (63, 64). In instances where neutrophils are unable to kill all the bacteria in these environments, the reactive oxygen species they release may trigger more capsule production, which could contribute to further invasion and systemic spread of Group 3 capsule-producing ExPEC. This may also occur in *G. mellonella* infections, in which hemocytes attempt to kill diverse bacteria in part through oxidation (65–67). In this model, the M12 *oxyR* mutant is severely attenuated (Fig. 3) because it is sensitive to oxidative stress and also unable to activate capsule production.

## MATERIALS AND METHODS

### Bacterial strains and conditions

M12 wild-type and mutant strains were routinely cultured at 37°C in Luria-Bertani (LB) medium, supplemented when needed with kanamycin (50 µg/mL), chloramphenicol (10 µg/mL), or ampicillin (100 µg/mL). The M12 transposon library was created as previously described (30).

### Blood and serum survival assay

Blood was collected from five healthy donors by venipuncture into serum-separating tubes and was centrifuged at 2,000 rpm for 15 min. The extracted normal human serum (NHS) was pooled, aliquoted, and stored at −80°C for further use. Heat-inactivated serum (HIS) was prepared by heating the NHS at 56°C for 30 min. Whole blood was also obtained from two healthy volunteers by venipuncture into sodium heparin tubes. C3-depleted human serum was obtained from Complement Technology, Inc. (Tyler, TX, USA). Cultures to be tested were subcultured, grown to the exponential phase, and standardized to 5 × 10^8^ CFU/mL. Serial dilutions were performed in phosphate-buffered saline (PBS), and 5 × 10^5^ CFU were mixed in triplicate 100 μL volumes of heparanized whole blood or serum. Samples were taken immediately (time 0) and again at 60 and 120 min, followed by serial dilution, plating, and incubation at 37°C overnight to enumerate bacterial survival. Survival percentages were calculated for time 60 and 120 by dividing the CFU/mL by their respective time 0 values.

### Percoll gradient separation of encapsulated and unencapsulated bacteria

The Percoll gradient separation method was adapted from reference 41. Solutions of 75% and 55% Percoll (1 mL each) were layered in polystyrene culture tubes. Then, 200 μL culture volumes containing ~1 × 10^9^ CFU (from either the M12 transposon mutant library or the wild-type parent M12 strain to isolate spontaneous mutants) were dispensed at the top of the gradient and centrifuged at 3,000 × *g* for 30 min at 6°C using a swing-bucket rotor. To recover unencapsulated populations from the bottom 75% gradient, the tube was sterilized with 70% ethanol and punctured with sterile tweezers. The bottom fraction of the gradient was collected, diluted, and spread on LB plates to recover individual colony-forming units. Individual transposon mutant colonies were screened for loss of capsule synthesis by alcian blue staining (see below) or by Percoll gradient separation. The transposon insertion sites for a small number of unencapsulated individual mutant colonies were determined by arbitrary PCR and Sanger sequencing (Eton Biosciences) as previously described (68).

### Tn-seq analysis

Three aliquots of the M12 transposon library were incubated for 2 h in LB and applied to Percoll gradients to separate the unencapsulated subpopulations, which were recovered and grown for 8 h before harvesting the DNA. The same aliquots were grown in LB but without Percoll separation as the control samples. The aliquots were then combined to generate pooled DNA representing unencapsulated or control bacteria. This entire process was performed twice to generate two independent samples for control and unencapsulated bacteria. Genomic DNA extraction, fragmentation, addition of Illumina adapters with barcodes, mapping to the M12 genome, analysis of raw count tables, and calculation of fold change values were performed as described previously (30). Illumina 100 or 150 bp reads were generated on a NextSeq 2000 P1 Illumina reads (single ends) at SeqCenter. Candidate genes required for capsule synthesis were identified by a log_2_-fold change >2 and a *P* value < 0.05. The list of genes in the M12 genome, their raw counts and reads per kilobase mapped for each sample, raw and adjusted *P* values for each gene, is shown in Table S1.

### Capsule isolation and Alcian blue staining

Capsules were released from pelleted cells by heat treatment (55°C for 30 min) and analyzed using polyacrylamide gel electrophoresis as previously described (28, 30). Negatively charged capsule in the gels was visualized by staining with 0.125% Alcian blue in 40% ethanol/5% acetic acid.

### Gene deletion and complementation

Primers used in creating defined mutants and complementation plasmids are listed in Table S4. Gene deletions were generated using the pFOK allelic exchange plasmid as described (69). Briefly, 500 base pair regions flanking the gene to be deleted were amplified and inserted with NEBuilder HiFi DNA Assembly (NEB) into EcoRI-linearized pFOK. Merodiploids were selected following conjugation with donor strain on LB agar plates containing 50 μg/mL kanamycin. Counterselection was performed at 30°C on no-salt LB plates with 20% sucrose and 0.5 μg/mL anhydrous tetracycline. Potential mutants were identified by colony PCR and verified by Sanger sequencing. Mutants were complemented by cloning the disrupted gene into a multicopy plasmid pJET1.2 (Thermo Scientific) or pUC19. Plasmids were sequence verified and transformed into the relevant mutant strain by electroporation.

### β-Galactosidase assay

Reporter plasmids were created by PCR amplification of the plasmid backbone and inserts, followed by Gibson assembly. Plasmid pTS1 contains the entire putative *kpsD* promoter, including the ribosome binding site and start codon, fused to *lacZ* in pTS12 (70). pTS3 was created from pTS1 using primers that exclude the putative OxyR-binding and −35/−10 sites. Site-directed mutagenesis was used to alter the OxyR-binding site in pTS1 to create pRDP3. The reporter plasmids were verified by Oxford Nanopore sequencing (Plasmidsaurus) and β-galactosidase levels were measured by Miller assay as described previously (71).

### *Galleria mellonella* infections

*G. mellonella* larvae were purchased from Best Bet, Inc. (Blackduck, MN, USA), stored at 15°C in the dark, and used within 2 weeks. An overnight culture of bacteria was subcultured and grown to an absorbance of 1.0 at 600 nm and diluted in PBS. Larvae without any evidence of melanization were injected through the left hindmost proleg with 10 μL of the inoculum containing 10^5^ CFU using a Hamilton 701RN syringe and a 30-gauge needle. Control larvae were injected with 10 μL of sterile PBS. The larvae were incubated at 37°C, and survival was monitored over a 48-h period.

### Isolation of spontaneous capsule mutants

To isolate spontaneous mutants, the bottom fraction from a Percoll separation was subcultured directly into fresh LB broth, grown to saturation, and the gradient separation was repeated before serial dilution and plating. Single colonies were screened for loss of capsule synthesis by Alcian blue staining. DNA from one unencapsulated colony from each experiment was isolated for whole bacteria genome sequencing. Illumina sequencing was performed (SeqCenter), and in some instances, long-read Nanopore sequencing (Plasmidsaurus) was performed to identify larger insertions or rearrangements. Polymorphisms were identified by mapping the Illumina reads to the complete M12 genome using Mapping and Find variations/SNP functions within Geneious software platform (Biomatters). Mutants with frameshift mutations in *kpsC* were tested for their ability to revert to capsule synthesis by inserting a barcoding plasmid (46) encoding chloramphenicol resistance to prevent contamination. The barcoded mutants were sequentially grown to saturation in LB broth containing chloramphenicol, followed by Percoll separation and recovering the top portion. After four passages, the top portion was diluted and plated, and single colonies were tested for capsule production. Whole-genome Illumina sequencing of putative revertants was performed by SeqCenter. The frameshift mutants and the putative revertants were also separately verified by PCR amplification and Sanger sequencing (Eton Biosciences).

### Phylogenetic analysis

Genomes similar to M12 were identified using the Similar Genome Finder Service at the Bacterial and Viral Bioinformatics Resource Center. The default settings were used (maximum 50 genomes returned, maximum distance of 0.05), and the list was manually curated to remove duplicate entries. Complete K96 *kps* loci were identified by BLASTN search at the National Center for Biotechnology Information using the M12 reference sequence. Sequences with >99.9% identity across the entire region were selected. Multiple sequence alignment was performed using the Geneious Prime application, and annotations of K96 genes were individually inspected to identify inactivating mutations.

### Statistical analysis

Survival of bacteria in blood and serum, β-galactosidase, and *Galleria mellonella* infection assay data were analyzed using GraphPad Prism 5.0. The statistical tests performed, as well as the significance values, are indicated in the individual figure legends.

## Data Availability

Raw reads for all transposon sequencing experiments and assemblies for spontaneous capsule mutants have been deposited in the SRA (PRJNA1474587).

## SUPPLEMENTAL MATERIAL

Supplemental Figures msphere.00237-26-s0001.pdf Fig. S1 and S2.

Supplemental Tables msphere.00237-26-s0002.xlsx Tables S1-S4.

ASM does not own the copyrights to Supplemental Material that may be linked to, or accessed through, an article. The authors have granted ASM a non-exclusive, world-wide license to publish the Supplemental Material files. Please contact the corresponding author directly for reuse.
