# Supplementary material for: Genetic control of Group 3 (K96) capsule synthesis and complement resistance in extraintestinal pathogenic Escherichia coli
Source: mSphere. 2026 Jul 10;11(7):e00237-26. doi: 10.1128/msphere.00237-26 (PMC13410964; doi:10.1128/msphere.00237-26)
Supplement: Supplemental Figures — Fig. S1 and S2. [file msphere.00237-26-s0001.pdf]

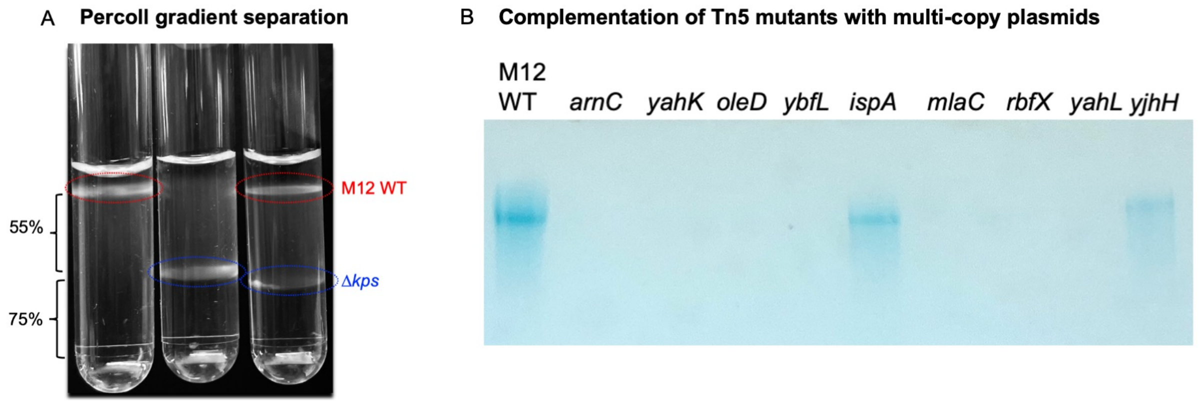

Supplementary Figure 1. Isolation of capsule mutants using Percoll gradient separations. (A) M12 wild-type (left), Δ*kpsCS* mutant (middle) and mixture of both strains (right) were applied to Percoll gradients (55% and 75%) to demonstrate separation of the encapsulated (top layer) from unencapsulated bacteria (bottom layer). Percoll gradients were used to isolate individual transposon mutants that do not produce capsule from an M12 Tn5 library (listed in Table 1). (B) *ispA* and *yjhH* are required for capsule synthesis. Unencapsulated Tn5 mutants that mapped outside of the *kps* region were complemented with plasmids containing the disrupted genes, and capsule production was assessed by Alcian blue staining. The gene that was disrupted and subsequently inserted in each case is shown above the lane.

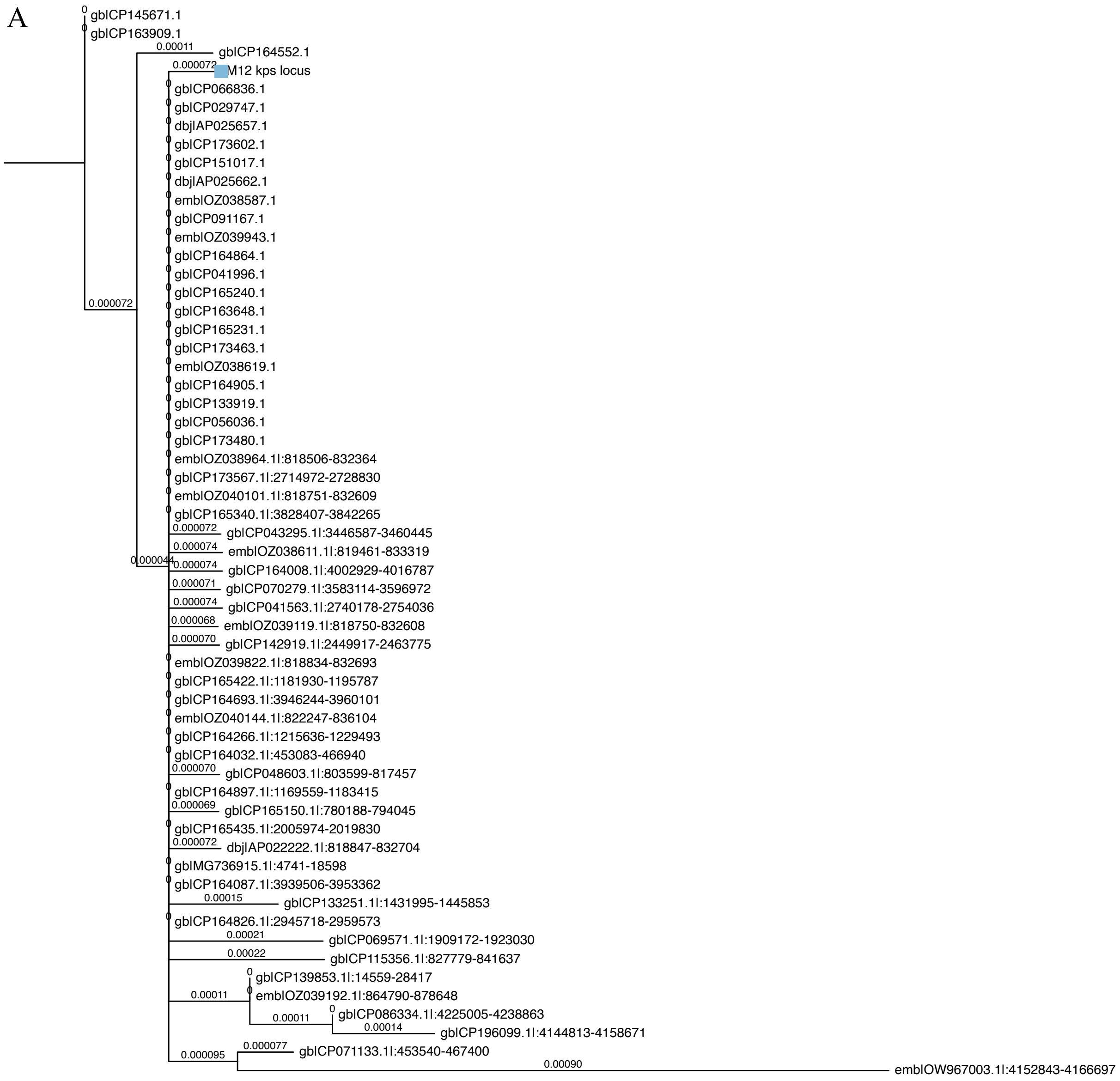

Supplementary Figure 2. Complete K96 loci in NCBI strains. Neighbor-joining tree of 58 K96 sequences with high nucleotide identity to M12. Accession numbers and genome regions containing the *kps* locus are indicated.
